# Assessment of arthroscopic shavers: a comparison test of resection performance and quality

**DOI:** 10.1186/s13018-020-01596-8

**Published:** 2020-02-21

**Authors:** Peng Liang, Gaiping Zhao, Xuelian Gu, Zhi Chen, Shaorong Xu, Weiguo Lai, Wentao Liu

**Affiliations:** 1 Department of Medical Instrument and Food Engineering, University of Shanghai for Science and Technology, Shanghai, China; 2 Shanghai BJ-KMC Medical Technology Co., Ltd., Shanghai, China; 3 Shanghai Ligetai Biotechnology Co., Ltd., Shanghai, China

**Keywords:** Shaver blades, Resection performance, Tensile and torsion tests, Corrosion resistance

## Abstract

**Background:**

Arthroscopic shavers play an indispensable role in arthroscopic debridement. They have exquisite structures and similar designs. The purpose of this study was to establish a reproducible testing protocol to compare the resection performance and the quality (tensile strength, torsional strength, and corrosion resistance) of different arthroscopic shavers with comparable designs. We hypothesized that there could be little difference in resection performance and quality between these shavers.

**Methods:**

Incisor Plus Blade (IPB; Smith & Nephew, Andover, MA) and Double Serrated Plus Blade (DSPB; BJKMC, Shanghai, China) were selected for resection performance and quality test. For resection performance testing, the resection torque, which is the minimum torque required to cut off silicone blocks with the same cross-sectional area, was measured to evaluate the resection performance of shaver blades when the other factors remain the same. For quality testing, tensile and torsion tests of the shavers’ joint part were performed, and ultimate failure load and maximum torque were measured and compared. The corrosion resistance of these blades was assessed by the boiling water test based on the ISO13402.

**Results:**

No significant difference existed in the resection torque between the shaver blades of IPB and DSPB (*P* = 0.54). To the failure load of shavers’ joint parts, IPB was significantly higher than DSPB, both in the outer and inner blades (*P* < 0.0001). The maximum torque of the joint part had no significant difference between IPB and DSPB (for inner blades *P* = 0.60 and outer blades *P* = 0.94). The failure load (for both IPB and DSPB *P* < 0.0001) and maximum torque (for IPB *P* = 0.0475 and DSPB *P* = 0.015) of the inner blades were higher than those of the outer blades. No blemishes were observed on the surface of the blades after corrosion resistance tests.

**Conclusions:**

This study provided some new methods to evaluate the resection performance and quality of different shavers. The resection performance, the torsional strength of the joint part, and the corrosion resistance of IPB and DSPB may show comparable properties, whereas the tensile strength of the shavers’ joint part showed some level of difference.

## Background

The development of arthroscopic surgery has dramatically changed the diagnosis and treatment of various joint diseases [1]. The shaver system plays an indispensable role in arthroscopic surgery. The shavers can clean the joint space by removing soft tissue such as the synovium, and even some of the denser tissues such as degenerated cartilage fragments and dissociated cartilage, as well as the trimming of the meniscus [2–4]. During the preparation for reconstructive surgery, arthroscopic shavers are used to clean the ligament residue and prepare for the reconstruction of the ligament [5]. Hence, the shaver is the most commonly used instrument by many arthroscopists [6].

**Fig. 1:**
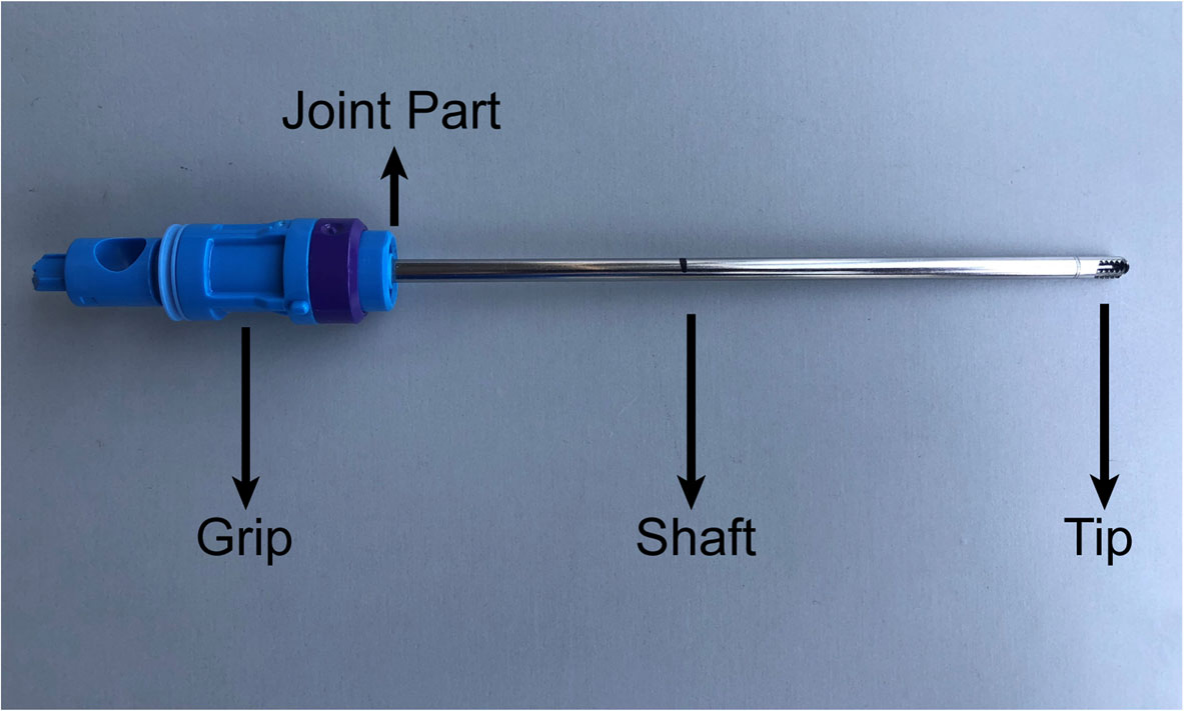
Different parts of a shaver blade

The shavers produced by different manufacturers vary, but certain structural compositions are common, such as a handpiece with the high-performance motor, arthroscopic shaver blades for cutting tissue, and a power source related to irrigation and suction. Pedals are mostly used to control the speed and direction of the blades. Shaver blades consist of grips, shafts, and tips (Fig.  1). The shafts and tips of the blades are mainly made of metal, while the grips are primarily made of composite plastic. This type of connection structure is sophisticated, and the failure mode is difficult to predict. Currently, different shavers are available in the market. They have various sizes (in length and diameter) and shapes (straight, curved, flexible) to meet diverse types of joint surgery. In addition, other features, such as the tooth and edge profiles, and the size of the cutting window, affect the sharpness of the blades and the performance of resection [7]. The cutting performance and the design of the surgical tool are as essential as the doctor’s experience and play a vital role in the success of the operation.

Few published studies have evaluated the performance and design of new shaver blades. King [8], Kobayashi [9], and Ledonio [10] have mainly investigated reprocessing of the shaver blades, including the level of contamination, the quality of the reprocessed blades (mechanical damage and sharpness), and the smoothness of the surface after resection. Moreover, there are almost no uniform test parameters and clear evaluation indicators in the test. Wieser et al. [11] performed the test in a controlled setting and pointed out that each blade has an optimal pressure and speed of rotation. This is one of the first established test protocols for the shaver systems in the literature, which is quantitative, independent, and repeatable. They also pointed out that the shaver blade with a smooth outer blade and serrated inner blade performed best for tendon resection. However, other parameters such as suction, contact pressure, and rotation speed, as well as some irrelevant variables such as the moisture on the surface of the tendon, were involved in this test, which may affect the accuracy of the experiment. In addition, no quality tests were performed on the parts other than the teeth of the blade.

The purpose of this study was to evaluate the resection performance and quality of different shavers using a series of quantitative, repeatable test methods. Specifically, tests were devised, and the resection performance and quality were evaluated by parameters such as resection torque, ultimate failure load, maximum torque, and corrosion resistance. We hypothesized that there could be little difference in resection performance and quality between shavers from two different manufacturers with comparable designs.

## Methods

This research mainly included two aspects of the assessments of different shavers: (1) The resection torque of the shaver blades was tested using a microcomputer-controlled electronic torsion tester (model CTT1501; MTS SYSTEMS, Shenzhen, China). As the magnitude of the cutting torque can reflect the performance of the shaver blades, it was also called the performance test. (2) The quality test of the shavers included three contents: tensile tests, torsion tests of the shavers’ joint part, and corrosion resistance of the blades.

### Shaver systems and blades

Tests were designed to quantitatively analyze and evaluate the performance and quality of two different shavers, Incisor Plus Blade (IPB; Smith & Nephew, Andover, MA) and Double Serrated Plus Blade (DSPB; BJKMC, Shanghai, China). The categories of shavers included devices that were (1) the Dyonics Power Shaver System (Smith & Nephew, Andover, MA), and (2) the Caines Shaver System (BJKMC, Shanghai, China). All packagings were intact, and there were no visible quality defects. Although the blades came from different manufacturers, they had comparable designs. Serrated feature on the inner and outer blades significantly affects the sharpness of the blades. Moreover, the performance of the blades was also affected by the size (length, diameter, cutting window) [7]. Hence, the sharpest (with serrated inner and outer blades) and the similar size shaver blades (IPB: 130 mm effective length and 4.5 mm diameter; DSPB: 130 mm effective length and 4.2 mm diameter) were chosen to avoid irrelevant variables in the performance evaluation.

### Performance testing

**Fig. 2:**
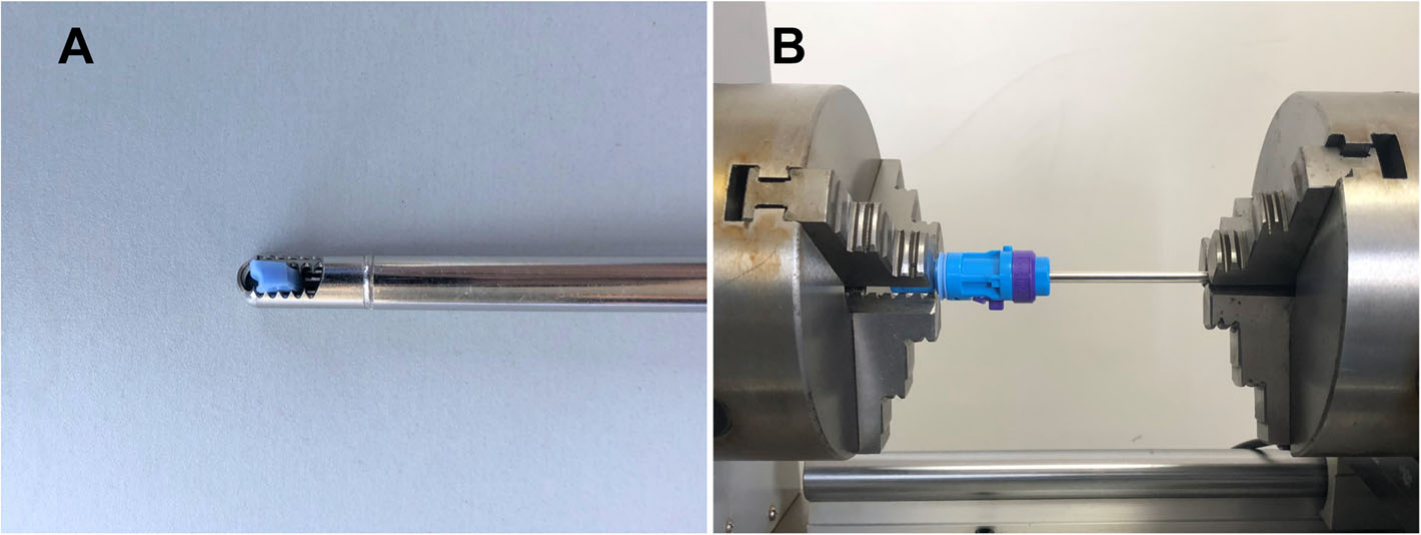
Performance test setting. **a** Silicone block position. Silicone blocks were used to compare resection performance. To avoid movement of the silicone block within the cutting window, the inner blade was slightly rotated to apply pre-tightening force. **b** Testing apparatus. The grip of the inner blade was fixed by the left clamp in a testing machine while the shaft of the outer blade was secured by the right clamp

The performance of shaver blades to resect soft tissue was evaluated by measuring the minimum torque required to cut off the silicone blocks with the same cross-sectional area. Silicones are commonly used as soft tissue simulants due to their consistency, durability, and comparable tissue density (approximately 1000 kg m^-3^) [12]. Therefore, in order to ensure the consistency and stability of the cutting object, silicone blocks with the same cross-sectional area were chosen as the model for soft tissue resection performance comparison. All silicone (Silicone Hardness: 50 Shore A; Hong Ye Jie Technology, Shenzhen, China) for testing was obtained from the local market. Silicone was cut into blocks with the same cross-sectional area (length 2.75 mm, width 1.75 mm), which was the model that mimicked soft tissue. The samples were placed vertically inside the cutting windows of the blades before testing. The inner blade was slightly rotated to apply pre-tightening force to avoid movement of the silicone block within the cutting window before resection (Fig. 2a).

First, ink marks were placed on the shaft of the blades, 80 mm from the tips. The grip of the inner blade with the silicone block was then positioned in the left clamp of the torsion testing machine (model CTT1501; MTS SYSTEMS, Shenzhen, China). The right clamp was adjusted to move horizontally so that the tip of the blade was inserted into the movable clamp without touching the silicone block and carefully moved to the previously marked reference position on the shaft. Then the shaft of the outer blade was mounted in the right jig (Fig. 2b). The torsion tester was driven so that the silicone block in the cutting window was cut off. After the cutting was completed, the peak torque was read and recorded, which was the minimum resection torque required to cut a unit volume of silicone block. Each resection torque test was performed using a new shaver blade and silicone blocks. Ten tests were performed for each different shaver blade for statistical comparison.

### Quality testing

#### Tensile and torsion testing

Cutting instruments with disposable blades, especially knives, not only may break but may become detached from the handle and be dropped free within the joint [13]. Therefore, tensile and torsion tests were performed to evaluate the quality of the joint part between the grip and the shaft of the shaver blade. Twelve new shaver blades were used, which were divided into 2 groups (with 6 blades per group, 3 from Smith & Nephew and 3 from BJKMC). Each blade was unwrapped and separated into the outer and inner blades before the test. Over 48 test cycles were performed. The tensile failure load and the ultimate torque, which were the indicators for evaluating the strength of the joint part, were recorded.

**Fig. 3:**
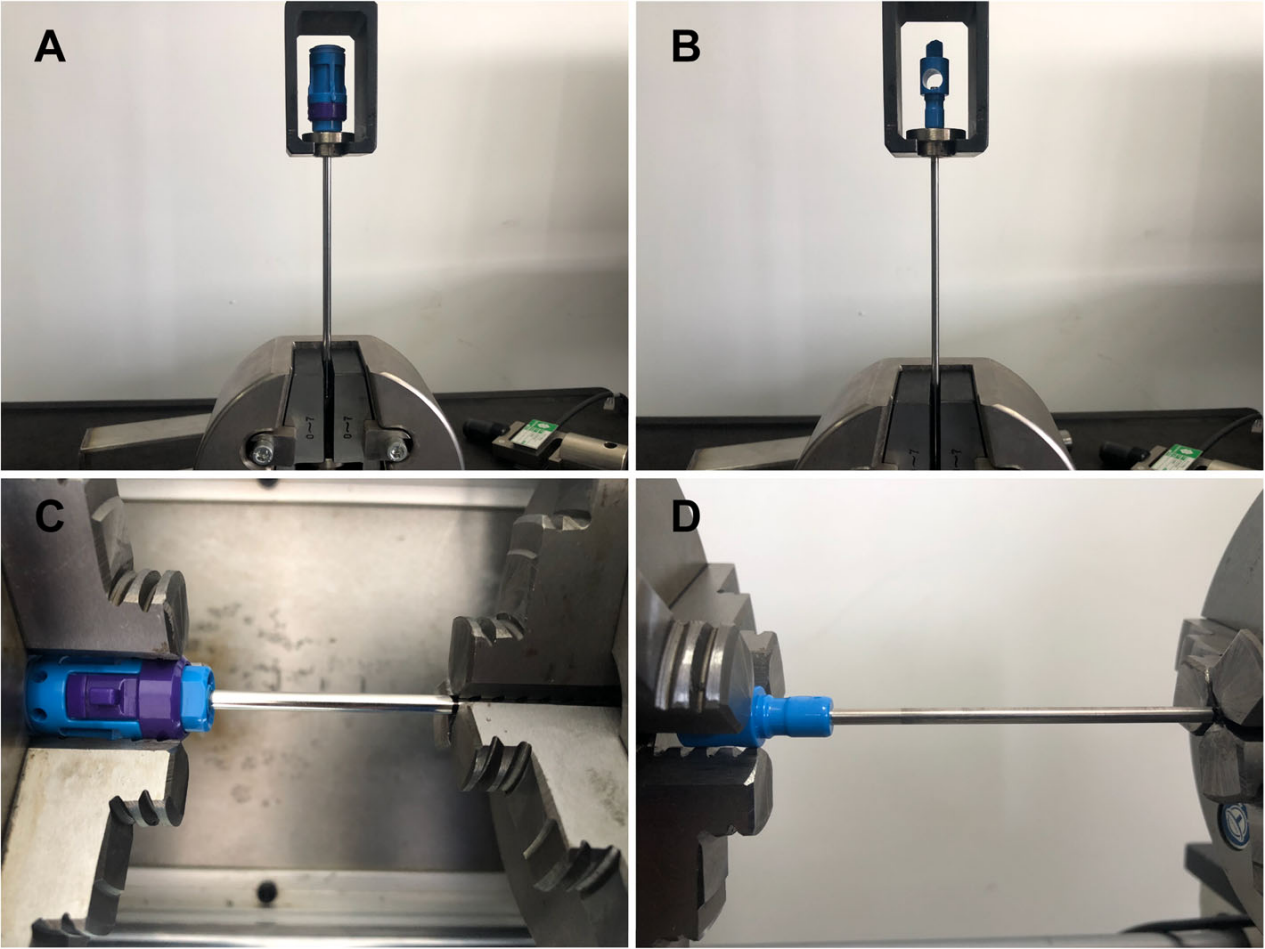
Quality test setting. **a** Tensile test of the outer blade. **b** Tensile test of the inner blade. **c** Torsion test of the outer blade. **d** Torsion test of the inner blade

In the first test session, the ability of the metal-to-plastic joint to resist tensile damage was investigated. The tensile tests were performed with a microcomputer control electronic universal testing machine (model WDW-5; HuaLong, Shanghai, China). In the first group, the blades were first passed through a custom-made metal ring, and then the blades were mounted with the metal ring onto a custom-designed steel holding device. The grips of the shaver blades were fixed in the upper actuator arm with the metal ring and the holding device. The shaft of the shaver blades was fixed to the clamp on the base platform (Fig. 3a). By adjusting the clamp and the upper arm of the test machine, this system allowed the axis of the shaver blades to be positioned in line with the applied tensile load (Fig. 3a and b). Then, a preload of 20 N was applied after there was no load on the blades. After preloading, the blades were tested to failure at 200 mm/min with load application in the axis of the shaft. Load-displacement curves and the load to failure were recorded.

The second test session was performed to simulate real use conditions and investigate the ultimate torsion of the joint. Briefly, the strength of the joint part was defined as the ultimate failure load during tensile loading and the maximum torque during torsional loading. The torsion tests were performed on an electromechanical torsion testing machine (model CTT1501; MTS SYSTEMS, Shenzhen, China). First, the grip of the blades was inserted into the left clamps’ chucks in the test machine. The clamp was tightened to ensure that the grip was securely attached to the test machine. The clamp of the right side was moved horizontally to the position of the specified mark, and then the shaft of the blade was tightened down as well by the right clamp (Fig. 3c and d). The torsion testing machine was driven to increase the torque at 5 rpm until the joint broke. The torque angle curve was recorded. Each test was performed 3 times (including inner and outer blades). The ultimate torque was collected, and the average torque was calculated for statistical analysis.

#### Corrosion resistance testing

The shaver blades were tested for corrosion resistance by boiling water test, which was established according to ISO 13402 [14], Surgical and dental hand instruments—Determination of resistance against autoclaving, corrosion and thermal exposure. The boiling water test is specified for determining corrosion resistance, and it should be noted that the water used in all tests shall be of quality 3 in accordance with ISO 3696 [15]. For this part of the research, 10 new shaver blades from 2 manufacturers were used. The 10 new blades were immersed in a mixed solution of soap and trisodium phosphate at 60 °C for 10 min at room temperature. The blades were thoroughly rinsed with water and dried. All the blades were sequentially immersed in boiling water in the beaker for 30 min and cooled for 1 h in the cold water. Then the blades were taken out of the water and exposed to the air for 2 h. After that, the surfaces of the blades were wiped vigorously with a dry cloth. Subsequently, morphological analysis (detection of corrosion marks on metal surfaces) was performed. Each surface was assessed for blemish, with any visible imperfections being recorded. According to the corrosion marks on the surface of the blades, corrosion resistance was divided into the following four grades: (A) Without any marks of corrosion, (B) There are slight marks of corrosion that can be removed by wiping. (C) There are marks of corrosion that cannot be removed by wiping. (D) There are serious corrosion marks that cannot be removed by wiping. All of the blades were evaluated and recorded for corrosion resistance levels.

### Statistical analysis

Statistical analysis was performed with GraphPad Prism software, version 7.0 (GraphPad Prism, San Diego, CA). The Student *t* test was used to detect any significant differences in resection torque, failure load, and maximum torque between different manufacturers’ blades. Statistical significance was present if a probability level of *P* < 0.05.

## Results

### Performance testing

**Fig. 4:**
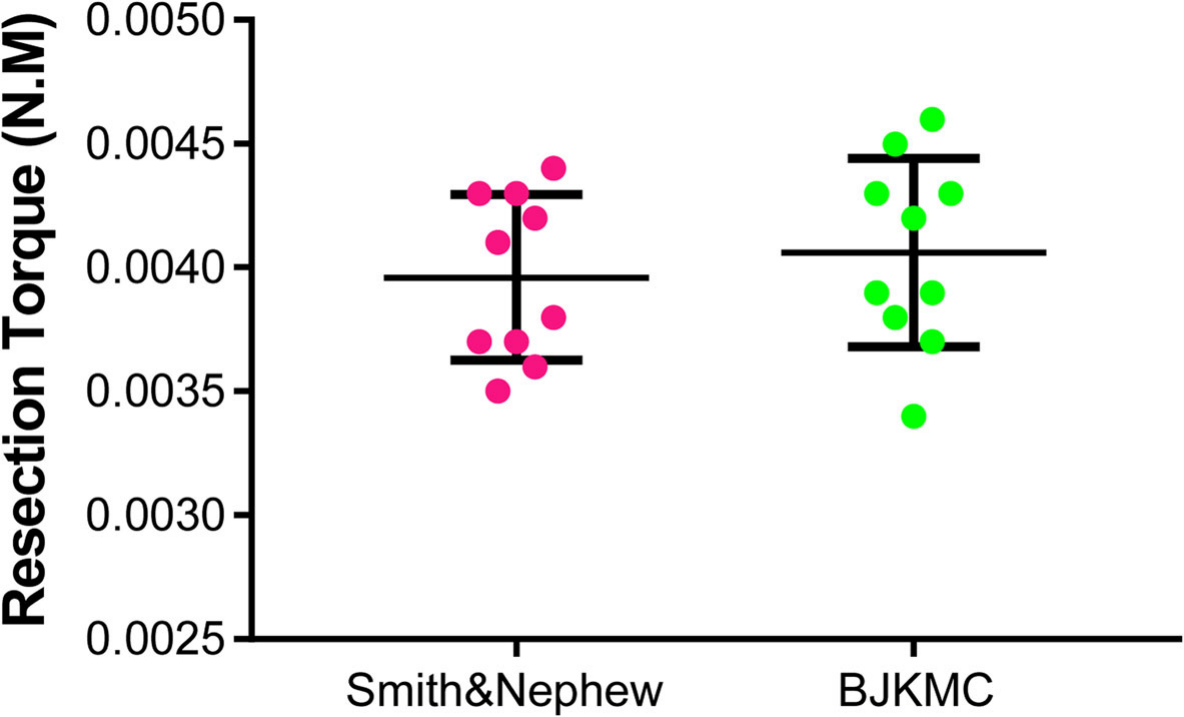
Resection torque of IPB and DSPB. No statistically significant differences were demonstrated in the resection torque. *IPB* Incisor Plus Blade (Smith & Nephew), *DSPB* Double Serrated Plus Blade (BJKMC)

The resection torque of two similarly designed shaver blades is presented in Fig. 4, and the resection torque data are listed in Table 1. No statistical difference existed in the resection torque between IPB (0.00396 ± 0.00033 N·m) and DSPB (0.00406 ± 0.00038 N·m;*P* = 0.54).

**Table 1:** Resection torque of shaver blades (N·m)

Test trial	IPB	DSPB
Test 1	0.0041	0.0034
Test 2	0.0037	0.0039
Test 3	0.0043	0.0045
Test 4	0.0037	0.0038
Test 5	0.0036	0.0043
Test 6	0.0035	0.0043
Test 7	0.0044	0.0037
Test 8	0.0038	0.0039
Test 9	0.0042	0.0046
Test 10	0.0043	0.0042
Mean	0.00396	0.00406
SD	0.00033	0.00038

*IPB* Incisor Plus Blade (Smith & Nephew)*, DSPB* Double Serrated Plus Blade (BJKMC)

The Student *t* test revealed no significant difference in resection torque between IPB and DSPB (*P* = 0.54)

### Quality testing

#### Tensile and torsion testing

**Fig. 5:**
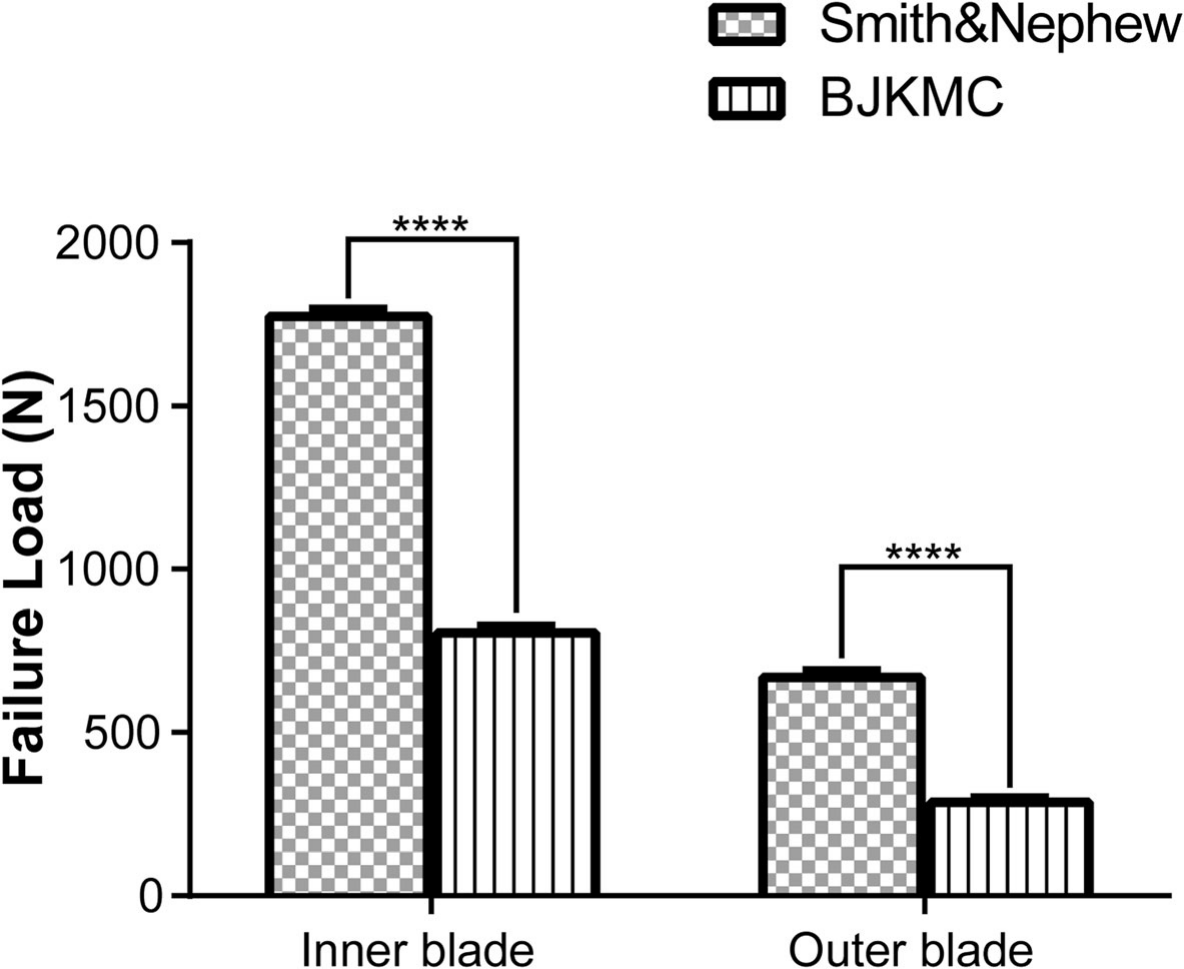
Failure load on the metal-plastic joint part of the blades. The bar chart shows there are significant differences in the failure load of the inner and outer blades between IPB and DSPB. The asterisks indicate statistically significant differences. *IPB* Incisor Plus Blade (Smith & Nephew), *DSPB* Double Serrated Plus Blade (BJKMC)

For tensile tests, the failure load of the shaver blades is illustrated in Fig. 5. The mean ultimate failure load of IPB and DSPB ranged from 805.7 N (DSPB) to 1773.9 N (IPB) on the inner blades and 286.4 N (DSPB) to 669.3 N (IPB) on the outer blades. The failure load of the IPB was significantly higher than DSPB, whether it is in the inner blades or the outer blades (*P* < 0.0001). Although the tensile failure load of Smith & Nephew devices is more than twice that of BJKMC products, the tensile strength of the joint part may have little effect on the cutting performance. The failure load of inner blades was higher than those of outer blades (for both IPB and DSPB *P* < 0.0001).

**Fig. 6:**
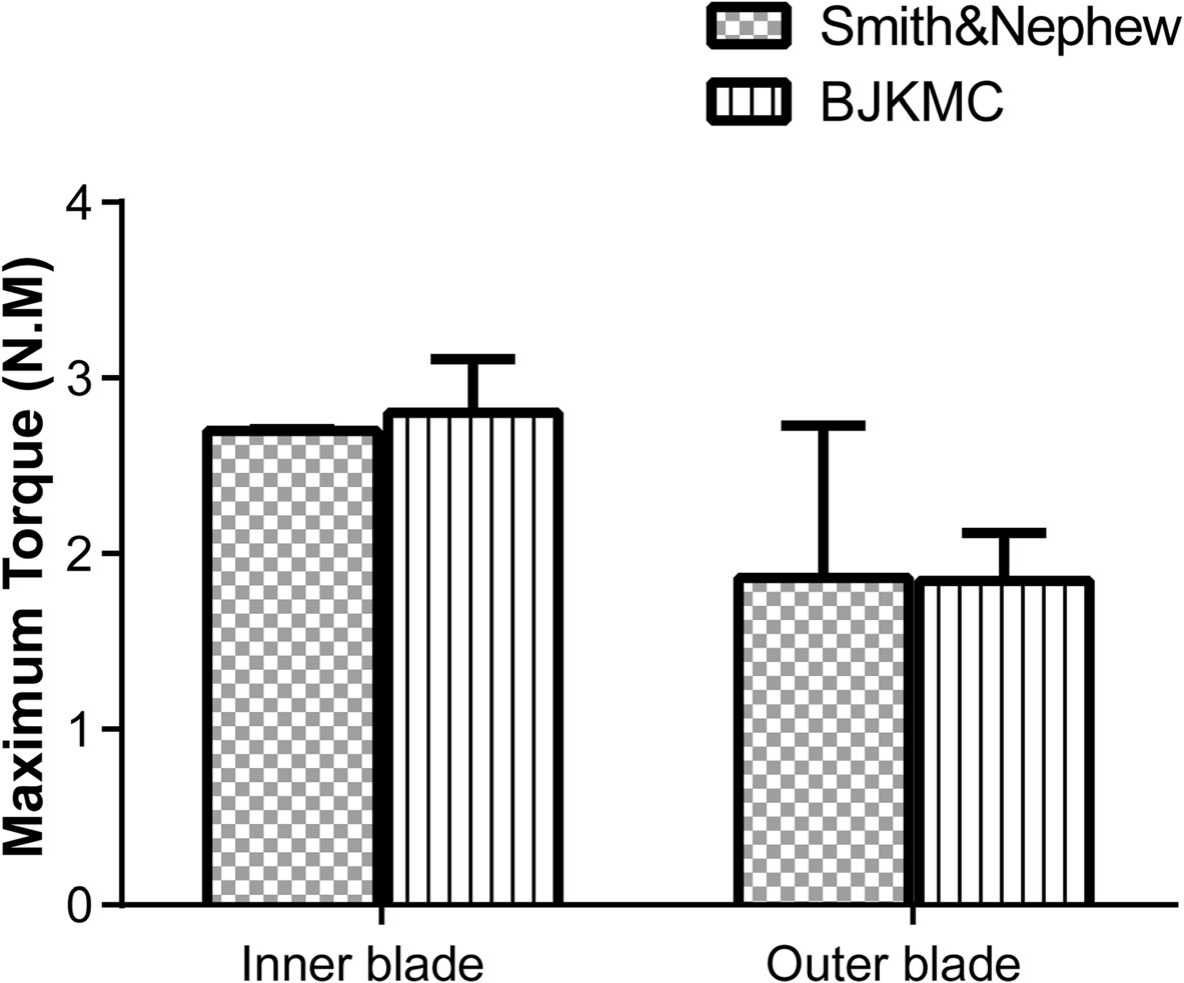
Maximum torque on the metal-plastic joint part of the inner and outer blades. No statistically significant differences were detected in maximum torque between IPB and DSPB. *IPB* Incisor Plus Blade (Smith & Nephew), *DSPB* Double Serrated Plus Blade (BJKMC)

For the torsion tests, the ultimate torque of two different shaver blades is depicted in Fig. 6. The average maximum torque of the BJKMC inner blade (2.8 ± 0.01 Nm) is slightly higher than the Smith & Nephew inner blade (2.7 ± 0.3 N·m; *P* = 0.60). For the outer blade, there is almost no difference in the maximum torque values (*P* = 0.94). In summary, the difference in the maximum torque between the two manufacturers’ shaver blades was not statistically significant (*P* > 0.05). The maximum torque of inner blades was higher than those of outer blades (for IPB *P* = 0.0475 and DSPB *P* = 0.015).

#### Corrosion resistance testing

**Fig. 7:**
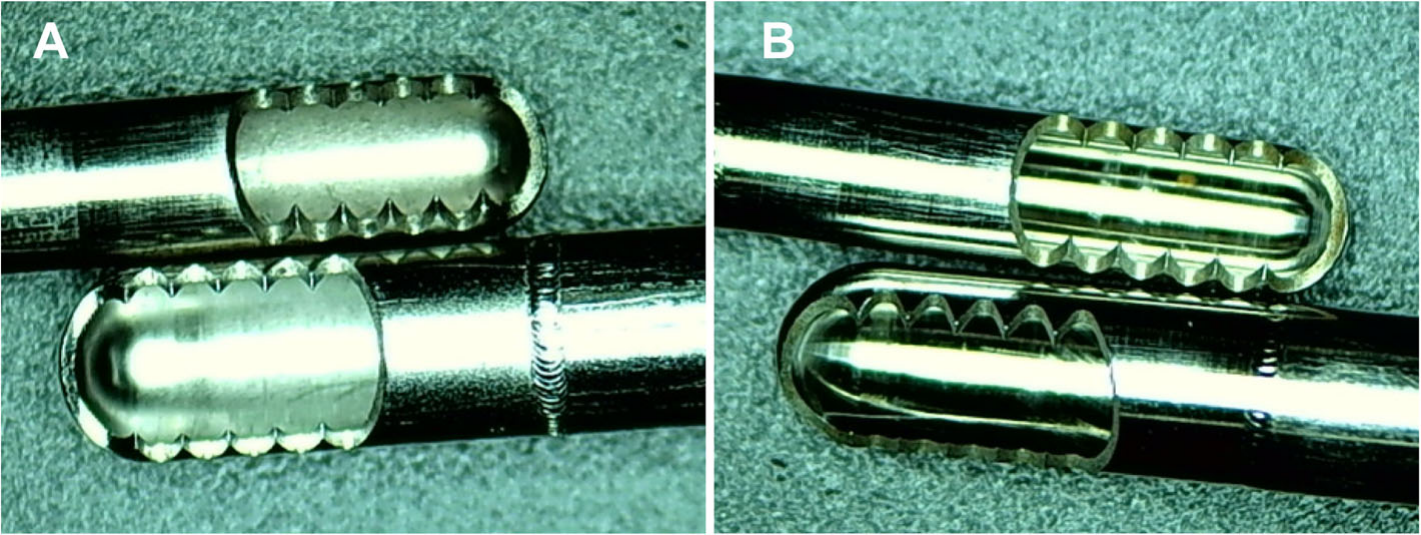
The surface appearance of the inner blades and the outer blades of Incisor Plus Blade and Double Serrated Plus Blade after the corrosion resistance test. **a** Smith & Nephew shaver blades. **b** BJKMC shaver blades

Figure 7 shows the appearance of the surfaces of the blades after boiling water testing. Each surface of the inner tube and the outer tube of the shaver blades was observed and assessed. No blemishes were found on the surface of the shaver blades through observation and morphological analysis, which meant that all the tested shaver blades in the corrosion resistance had reached the highest standard of class A.

## Discussion

Arthroscopic shaver system plays an indispensable role in arthroscopic surgery. Design and manufacturing of the shaver blades directly affect the resection efficiency of the soft tissue. Shaver blades with a wide range of cutting tips have been developed by the orthopedic instrument companies. They are designed for different clinical situations and specific functions [7]. However, the results of the relevant researches in the field of medical instruments such as cutting rate are lack of consistency because of the various parameters in the experiment, and the evaluation indexes are not yet clear [16]. We hypothesized that there could be little difference in the resection performance and quality between shaver blades from the two different manufacturers with comparable designs. Therefore, we tried to verify our hypothesis utilizing testing and testing data. Our initial hypothesis was partially supported by the results of the test. There is no statistically significant difference in the resection performance and torsion strength that could be detected among these blades. Furthermore, these shaver blades are indistinguishable in corrosion resistance. However, there was a significant difference in the tensile strength of the connecting part between the grip and the shaft among these blades of two different manufacturers as well as the inner blades and the outer blades.

To date, few known studies have directly evaluated the resection performance and quality of unused shaver blades, there is only one study on the testing of the new arthroscopic shaver systems from different manufacturers. Wieser et al. [11] performed a study to evaluate the performance of different shaver systems and blades and to explore the effect of speed and contact pressure on resection performance. They designed a holding device to control the contact pressure and then carried out a resection test on the soft tissue by comparing the weight of the cut bovine Achilles tendon per unit time. They did not detect any significant difference in resection performance between comparable blades of different manufacturers.

In previous studies, the resection performance of blades was generally evaluated by measuring the smoothness and depth of tissue damage [17] and the damage of the blades. However, morphological analysis cannot accurately reflect the quality of the cut tissue. Hence, the real surgical environment was simulated, and the cutting performance was evaluated by calculating the weight of the tissue before and after cutting per unit time [11, 18]. It is also difficult to precisely calculate the weight of the sample being cut because of many factors, such as different moisture content on the surface of the sample. A completely new approach was designed to compare resection performance. The torque required to cut a silicone block with a unit cross-sectional area was measured and the resection performance was assessed. The minimum resection torque required to cut the silicone block reflects the resection performance. The smaller the resection torque, the better the resection performance of the shaver. The results revealed no difference in the resection performance of the shaver blades from two different manufacturers, which is consistent with the results of prior studies by Wieser et al. [11].

The strength of the joint part must be ensured even if there are currently no cases of instrument failure caused by damage to the connection part. In Y et al. [19] presented a case of detachment of the tip of the motorized shaver within the knee joint. Gambardella and Tibone [20] reported a complication of blade missing from the knife handle within the knee joint. Although the incidence of these complications is low, additional surgery, such as arthrotomy, is required once it occurs, which will have an impact on the treatment and recovery of patients. Fortunately, the design of the three-piece blade (metal tip, plastic bar, and tube) is now entirely replaced by the design of the two-piece blade (metal bar and tube), which increases the integrity of the blade and reduces the incidence of complications of the detachment of the tip. However, most shaver blades are made up of shaft metal tubes (including cutting teeth) and plastic grips. Typically, the plastic–metal interface is a transition zone of high stress.

We performed quality tests by measuring the tensile and torsional strength of the joint part. However, the results of this part are contrary to our previous hypothesis. First of all, there is a significant difference in the tensile strength from different manufacturers. This is primarily caused by the fact that low requirements for tensile strength of the shavers during surgery. In order to ensure sufficient strength to avoid instrument failures during arthroscopic procedures, conservative designs are often used by manufacturers. In addition, the safety threshold of the joint strength is not known. It is necessary to establish the relevant standards in future studies.

Secondly, there are significant differences in the tensile strength and torsional strength of the inner and outer blades. It seems comprehensible that the tensile strength and torsional strength of the inner blades are much larger than the outer blades. When the shaver system works, the outer sheath remains stationary, playing the role of protecting the non-surgical part. The inner sheath cuts the soft tissue by rotating at high speed, and the tips of the blade acted as a two-edged. In general, the rate of removal in the oscillation rotation mode is higher than that in the clockwise and counterclockwise modes, which means that the inner blades need to be rotated frequently in an oscillating mode. Furthermore, the high rotational speed is applied to the inner blade to improve the efficiency of surgery. Therefore, in clinical use, the torsional strength of the joint part of the inner blade should be higher than that of the outer blade, which is reflected in the test results. Additionally, clogging is also a common complication during the cutting process. Clogging can be troublesome and may be kept to a minimum if suction is well regulated [21]. It seems that the higher torsional strength can ensure the integrity of the shaft and grip of the blades and reduce the incidence of instrument failures. Although the effect of tensile strength does not seem to be significant in terms of mechanism, the difference in tensile strength of the blades between different manufacturers cannot be ignored. In future studies, whether lower tensile strength and torsion strength increase the risk of device failure, and what is the threshold of achieving a sufficiently safe strength could be explored.

Most arthroscopic products are disposable, even though the reusing of shaver blades can save significant medical costs. Many studies have found that reprocessed arthroscopic shaver blades are at risk of quality defects and iatrogenic disease transmission. King tested 27 reprocessed single-use-only shaver blades and found that 48% had detectable levels of protein, 63% had detectable levels of nucleic acid, and all of the reprocessed blades had visual damage [8]. Kobayashi analyzed elements and chemicals of contaminants on reprocessed shaver blades and found that contaminations may signify a risk for disease transmission [9]. Wiser found that both soft tissue and bone resection resulted in a blunt shaver blade over time [11]. Smith confirmed that retained bone and other biodebris inside orthopedic instruments may not be sterile despite steam sterilization [22].

The importance of corrosion resistance cannot be ignored even though the time of immersion of the shaver blade in the solution is much short compared to that of an orthopedic implant. Pedowitz demonstrated that metallic micro debris is liberated by common arthroscopic instruments, and may induce intra-articular damage if their concentrations reach clinically significant levels [23]. In order to ensure good visualization, which is one of the key importance in arthroscopic surgery [24], arthroscopic surgery is usually performed with pressurized saline. The liquid pressure can enlarge the available joint space, and the liquid flow not only flushes out the joint-free tissue such as synovial or cartilage debris but also avoids thermal damage inside the joint [25, 26]. In other words, the shaver blades are in a closed pressurized physiological saline solution during operation. Hence, the shaver blades were tested for corrosion resistance by boiling water test. The results of this study show that the corrosion resistance of the shaver blades of two different manufacturers can reach grade A after the boiling water test. It should be noted that the corrosion resistance test is rough and basic; electron microscopy was not used to detect corrosion defects. The extent to which corrosion resistance affects resection performance is unknown, and this investigation is beyond the scope of this study.

There are limitations to this study. Only two similar products from different manufacturers were tested. In future research, many other manufacturers’ products could be tested. Silicone was chosen instead of animal tissue as a model for soft tissue resection performance comparison. However, the properties of medical-grade silicone match those of human tissues, and some scholars have used silicone materials to simulate tissue biomechanics [27]. An additional limitation of this study is that only the resection performance of the shaver blades was evaluated, and the performance of the whole shaver systems was not tested. Therefore, the real surgical environment was not simulated, and the resection test was not performed under closed hydrostatic pressure. Although simulating the operating environment will add many variables that may result in inaccurate test results, it will be the subject of future testing. Although there may be a significant difference in the tensile strength of the joint part, it is not known what the threshold of the tensile strength is. In future studies, the strength threshold required for the joint part of shaver blades under different clinical tasks will be evaluated to establish uniform standards among different manufacturers.

## Conclusion

The resection performance and the torsional strength of the shaver blades from two different manufacturers may show comparable properties. However, there is a significant difference in the tensile strength of the joint part between the different manufacturers’ shaver blades. The inner blades showed higher tensile strength and torsional strength than the outer blades. None of the shaver blades that were visually evaluated showed any blemish after the corrosion resistance test.

## Data Availability

None

## Abbreviations

DSPB: Double Serrated Plus Blade
IPB: Incisor Plus Blade

## References

[1] Macmull S, Gupte CM (2015). (ii) Basic knee arthroscopy: a brief history, surgical techniques and potential complications. Orthopaedics Trauma..

[2] Graf BK, Jr CW (1987). Motorized arthroscopic instruments: a review. Arthroscopy..

[3] Lui TH (2015). Endoscopic resection of lateral synovial cyst of the knee. Arthrosc Tech..

[4] Yeo N, Younger A, Veljkovic A, Waly (2019). Ankle arthroscopy: osteoarticular procedures. Arthroscopy and Endoscopy of the Foot and Ankle.

[5] Denti M, Quaglia A, Randelli P (2016). Arthroscopic anterior cruciate ligament reconstruction with bone-patellar tendon-bone. Arthroscopy.

[6] Tingstad EM, Spindler KP (2004). Basic arthroscopic instruments. Oper Tech Sports Med..

[7] Singh S, Tavakkolizadeh A, Arya A, Compson J (2009). Arthroscopic powered instruments: a review of shavers and burrs. Orthop Trauma..

[8] King JS, Pink MM, Jobe CM (2006). Assessment of reprocessed arthroscopic shaver blades. Arthroscopy..

[9] Kobayashi M, Nakagawa Y, Okamoto Y, Nakamura S, Nakamura T (2009). Structural damage and chemical contaminants on reprocessed arthroscopic shaver blades. Am J Sports Med..

[10] Ledonio GT, Arendt A, Adams E (2014). Reprocessed arthroscopic shavers: evaluation of sharpness and function in a cadaver model. Orthopedics..

[11] Wieser K, Erschbamer M, Neuhofer S (2012). Controlled laboratory testing of arthroscopic shaver systems: do blades, contact pressure, and speed influence their performance. Arthroscopy..

[12] Payne T, Mitchell S, Bibb R, Waters M (2014). Initial validation of a relaxed human soft tissue simulant for sports impact surrogates. Procedia Engineering..

[13] Philips BB, Canale ST, Beaty J (2012). General principles of arthroscopy. Campbell’s operative orthopaedics.

[14] International Organization for Standardization (1995). ISO 13402:1995 Surgical and dental hand instruments—determination of resistance against autoclaving, corrosion, and thermal exposure.

[15] International Organization for Standardization (1987). ISO 3696:1987 Water for analytical laboratory use—specification and test methods.

[16] Chen Z, Wang C, Jiang W (2017). A review on surgical instruments of knee arthroscopic debridement and total hip arthroplasty. Procedia Cirp..

[17] Green LM, King JS, Bianski BM, Pink MM, Jobe CM (2006). In vitro effects of 3 common arthroscopic instruments on articular cartilage. Arthroscopy..

[18] Ferguson BJ, DiBiase PA, D’Amico F (1999). Quantitative analysis of microdebriders used in endoscopic sinus surgery. Am J Otolaryngol..

[19] In Y, Bahk WJ, Park JB (2003). Detachment of the tip of a motorized shaver within the knee joint: a complication of Arthroscopic Surgery. Arthroscopy..

[20] Gambardella RA, Tibone JE (1983). Knife blade in the knee joint: a complication of arthroscopic surgery: a case report. Am J Sports Med..

[21] Strobel MJ, Strobel MJ (2002). Surgical instruments. Manual of arthroscopic surgery.

[22] Smith K, Araoye I, Gilbert S (2018). Is retained bone debris in cannulated orthopedic instruments sterile after autoclaving?. Am J Infect Control..

[23] Pedowitz RA, Billi F, Kavanaugh A (2013). Arthroscopic surgical tools: a source of metal particles and possible joint damage. Arthroscopy..

[24] Muellner T, Menth-Chiari WA, Reihsner R, Eberhardsteiner J, Engebretsen L (2001). Accuracy of pressure and flow capacities of four arthroscopic fluid management systems. Arthroscopy..

[25] Tuijthof GJ, Dusee L, Herder JL, van Dijk CN, Pistecky PV (2005). Behavior of arthroscopic irrigation systems. Knee Surg Sports Traumatol Arthrosc..

[26] del Piñal F (2011). Dry arthroscopy and its applications. Hand Clin..

[27] Sparks JL, Vavalle NA, Kasting KE (2015). Use of silicone materials to simulate tissue biomechanics as related to deep tissue injury. Advances in skin & wound care..

